# Pervasive sublethal impacts of heat exposure revealed by telomere dynamics

**DOI:** 10.1098/rspb.2025.2877

**Published:** 2026-09-16

**Authors:** Justin R. Eastwood, Niki Teunissen, Michelle L. Hall, Sjouke A. Kingma, Michael J. Roast, Nataly H. Aranzamendi, Ian R. Hoppe, Ariana M. L. Porte, Simon Verhulst, Anne Peters

**Affiliations:** ^1^ School of Biological Sciences, Monash University, 25 Rainforest Walk, Clayton, Victoria 3800, Australia; ^2^ Behavioural Ecology Group, Department of Animal Sciences, Wageningen University and Research, De Elst 1, 6708 WD Wageningen, The Netherlands; ^3^ Max Planck Institute for Ornithology, Vogelwarte Radolfzell, Schlossallee 2, 78315 Radolfzell, Germany; ^4^ Bush Heritage Australia, 395 Collins Street, Melbourne, Vic 3000, Australia; ^5^ School of Agriculture and Environment, University of Western Australia, Crawley, WA 6009, Australia; ^6^ Konrad Lorenz Institute of Ethology (KLIVV), University of Veterinary Medicine Vienna, 1a Savoyenstrasse, 1160 Vienna, Austria; ^7^ Groningen Institute for Evolutionary Life Sciences, University of Groningen, Nijenborgh 7, 9747 AG Groningen, The Netherlands

**Keywords:** climate warming, telomere length, early life, fitness, habitat degradation

## Abstract

Predicting species responses to anthropogenic environmental change remains one of the most pressing and complex challenges in contemporary biology. While mass mortality events and localized extinctions have been linked to global warming, the more elusive and probably common, effects of sublethal heat are less well understood. In a cooperatively breeding bird from the Australian tropical savannah, we identified that hotter conditions accelerated telomere attrition through two critical life-history stages: juveniles transitioning to nutritional independence and adults transitioning into a reproductive phase. In both stages, there was no capacity for environmental or social conditions to mitigate heat-related telomere attrition, except in adults inhabiting high-density vegetation. These findings demonstrate that sublethal heat effects can have pervasive cryptic consequences for individual somatic state, thereby reducing fitness. More generally, our findings, although probably widespread and concerning for population persistence, also highlight the importance of preserving climate refugia in conservation strategies amid rising sublethal temperatures.

## 1. Background

Climate warming is increasing the exposure of wildlife to sublethal heat [1], leading to cryptic physiological costs that may subsequently threaten lifespan or reproductive success [2–5]. If these sublethal heat effects are chronic and go uncompensated, they can reduce fitness and subsequently threaten population viability [6]. While not as immediate as mass mortality events [7], these sublethal effects potentially have a larger impact in nature and may have contributed to reduced population sizes and local extinctions already associated with climate warming [8,9]. Yet, despite these profound implications, the role of sublethal heat exposure in influencing wildlife responses to climate warming and population dynamics is less well understood.

Birds serve as a useful sentinel species group to detect the impact of anthropogenic changes on ecosystems owing to their sensitivity to climate [10]. Previous studies of birds have shown that exposure to thermally challenging environments is associated with increased metabolism, hyperthermia and dehydration; these can affect growth, body condition, performance behaviours and breeding success. Although important, such effects are often measured on a short timescale and are not necessarily representative of long-term, cumulative or chronic responses to sublethal heat. Furthermore, the effect of sublethal thermal conditions is also likely to depend on among-individual variability in responses and complex biotic and abiotic interactions that modulate heat exposure. For instance, physiological maturity and life-history [11], behaviour [12] or environmental conditions [13,14]. Thus, determining the overall cost of high temperatures requires high-quality ecological and individual data, as well as an integrated biomarker of the sublethal effects of heat exposure.

Telomeres are repetitive nucleoprotein structures at the ends of chromosomes that can serve as useful biomarkers of environmental stressors [15] and long-term survival [16,17]. These regions play a vital role in maintaining DNA integrity, and shorten during cellular replication [18,19] and in response to oxidative stress [20]. Consequently, over an individual's lifespan, telomeres become shorter with age [21] and act as a cumulative measure of lifetime cellular renewal and stress exposure [22,23]. In addition, higher telomere attrition rates have been linked with reduced survival and lifespan, especially when measured early in life [24]. These findings indicate that surviving poor early-life conditions may come at the expense of somatic maintenance and long-term survival [23]. Therefore, faster telomere attrition early in life and associated fitness costs could also suggest that exposure to sublethal conditions might have a lower impact later in life. In birds, telomere length tends to be shorter in response to poor health or malnutrition, high social competition, high reproductive effort, lower individual quality and unfavourable environmental conditions [15], including thermal conditions [25]. Heat exposure tends to be negatively [26,27] or nonlinearly [28] associated with telomere length. However, some studies show that rainfall or unknown factors could ameliorate costs in some species [26,29]. Yet other factors, such as habitat quality, social group conditions, water availability and individual factors, are rarely considered, despite their potential to buffer or exacerbate heat exposure. An integrative approach, combining how these various biotic and abiotic factors interact to modulate the effects of thermal conditions on telomere attrition, is therefore important to understand the effect of sublethal temperatures on wildlife.

Here, we investigate the impact of sublethal heat in a cooperatively breeding bird from the Australian tropical savannah, the purple-crowned fairy-wren (*Malurus coronatus coronatus*). Purple-crowned fairy-wrens are frequently exposed to sublethal hot conditions (annual mean ± s.d. of 36.6°C ± 3.8°C and 78 ± 27 days above 40°C per annum), further adding to other threats to population viability, including habitat degradation and loss. Previously, we demonstrated that purple-crowned fairy-wren nestling telomere length was shorter under hot, dry conditions, suggesting that sublethal heat exposure early in life may have an impact on Darwinian fitness [26]. Here, we further advance our understanding of sublethal fitness costs by exploring whether heat impacts vary across distinct life-history stages and which biotic and abiotic factors modulate those impacts under natural conditions. Across a 15-year individual-based field study, we assessed (i) telomere change across two biologically distinct and critical life-history stages; juveniles transitioning to nutritional independence and adults transitioning into a reproductive phase; (ii) whether the effect of sublethal heat on telomere change varies during those life stages; and (iii) whether heterogeneous individual, environmental and behavioural conditions can drive the cost of sublethal heat exposure. This study contributes to our understanding of how species are affected by rising sublethal temperatures and highlights the importance of modulating factors in mitigating those effects.

## 2. Material and methods

### (a) Study species and fieldwork

The purple-crowned fairy-wren *M. coronatus coronatus* is an Endangered passerine bird found along waterways of the monsoonal savannah of northwest Australia [30]. The climate in this tropical region is characterized by high rainfall and high temperatures during the wet season (November to April) and milder, low rainfall conditions during the dry season (May to October). Purple-crowned fairy-wrens inhabit riparian environments densely vegetated by *Pandanus aquaticus*. Breeding activity is heightened after rainfall, with a peak in activity during the wet season, although breeding may also occur throughout the year [31]. Breeding pairs are socially and sexually monogamous, are cooperative breeders and maintain year-round territories with 0–9 subordinate male and female helpers [32]. Dominant breeding pairs are identifiable in the field by unique duetting performances and nesting activity [33]. Helpers may assist the dominant pair with nestling provisioning and territory defence behaviours (against predators and intra-specific intruders) [34], which has been shown to benefit female reproduction and survival [35]. Females lay between two and four eggs (median of three) and incubate them for approximately 13 days. After hatching, nestlings fledge at approximately 13–14 days but remain dependent on parental care until approximately 56 days, after which they transition into independent subadults (approx. 90 days) [31]. After four months, individuals may start to disperse from their natal territory and move into either a subordinate helper or dominant breeding position elsewhere; alternatively, some individuals become subordinate or inherit a dominant breeding position on their natal territory. Dispersal and first-time breeding may occur after 2 years (16% of individuals). However, these events are outside the time limits of this study because of the reduced number of individuals repeatedly sampled during this later period.

**Figure 1.**
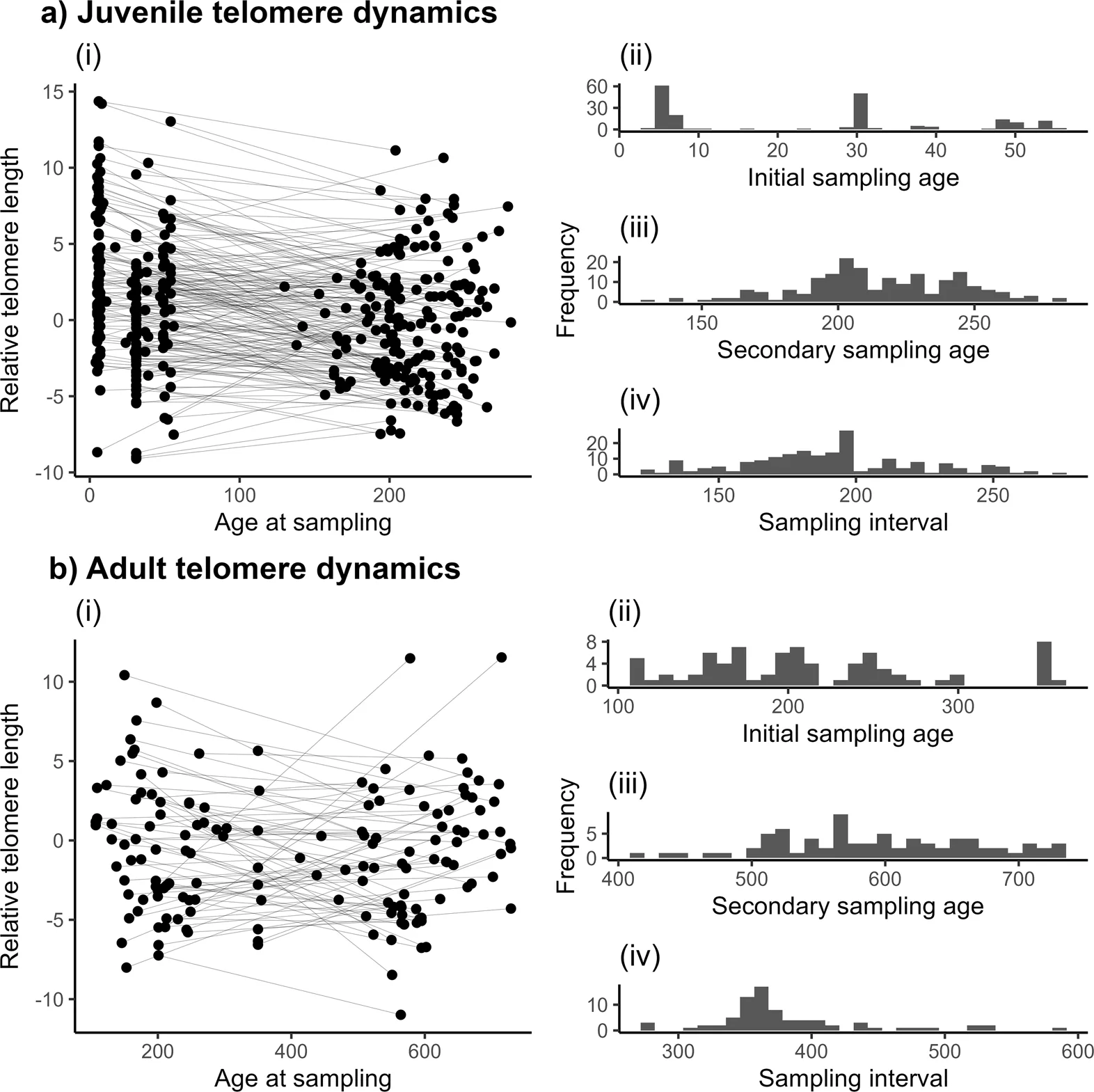
Study design and sampling regime across two distinct and biologically important life stages of purple-crowned fairy-wrens: (a) *Juvenile stage:* 192 individuals were sampled for telomere length when they were nutritionally dependent on parental care (nestling or fledgling age; mean = 24 days (s.d. = 18), range = 4–56 days) and then again after a 90-day climate window spanning the transition from dependent juvenile to independent subadult (age mean = 215 days (s.d. = 29), range 130–281 days; sampling interval mean = 190 days (s.d. = 32), range = 124–274 days). (b) *Adult stage:* 77 individuals were sampled for telomere length as independent subadults (age mean = 213 days (s.d. = 69), range = 107–355 days) and then again in their second year, which spans the transition from subadult to adult when individuals may disperse or gain a breeding position (age mean = 594 days (s.d. = 75), range = 413–729 days; sampling interval mean = 380 days (s.d. = 58), range = 275–584 days). Shows (i) individual telomere length at each sampling point, (ii) frequency of the age the initial measure was collected, (iii) frequency of the age the second sample was collected, and (iv) the sampling interval between the initial and second capture. Climate window intervals were standardized to 90 days for juveniles and 365 days for adults and began on the initial telomere sample date.

Our study population is located at the Australian Wildlife Conservancy's Mornington Wildlife Sanctuary in Western Australia (17°31′ S, 126°6′ E), along a 15 km stretch of Annie Creek and Adcock River. From 2005 to 2023, individuals were banded with a unique combination of a multicoloured band and a coloured numbered metal band from the Australian Bird and Bat Banding Scheme. Individuals were caught as nestlings with known hatch dates or fledglings, which could be accurately aged based on morphological characteristics and plumage colouration (median age error: 0.23 months). Young birds are identifiable by distinct plumage characteristics and moult into their sexually dimorphic adult plumage, with males having distinct black and purple head plumage [36]. Most adults are highly philopatric and stay or disperse within the core study area; additionally, we found dispersers in landscape-scale surveys [37]. This allowed us to recapture individuals for resampling. Blood was collected from the brachial vein using a 25g needle and a heparinized capillary tube for downstream molecular assays. The blood was centrifuged, and the plasma was removed. Between 2007 and 2011, red blood cells were stored in Longmire's lysis buffer and, afterwards, in 100% ethanol [38]. All samples were stored at 4°C until laboratory analysis. The sex of the birds was identified by sexually distinct plumage characteristics or a molecular assay previously adapted for use in this species [26,39].

### (b) Study design

We partitioned our analysis into two distinct and biologically important life stages, known to vary in telomere dynamics, physiology and social environment (figure 1). Climate windows (90 days for juvenile and 365 days for adult; figure 1) and sampling intervals were chosen *a priori* based on the criteria below and selected from a long-term dataset spanning 17 years (2007–2023). For inclusion, individuals must have been captured and sampled at least twice for telomere length, once before and once after the life-history period of interest. Owing to the haphazard nature of sampling wild birds, individuals vary in age at sampling and sampling intervals, which are controlled for in each statistical model. Climate windows were not calculated between the first and second capture because of the bias introduced by sampling interval variation combined with year-round breeding (i.e. longer intervals are always closer to the annual mean). Rather, each climate window began at the initial capture life-history stage and ended after 90 and 365 days for juveniles and adults, respectively.

**Figure 2.**
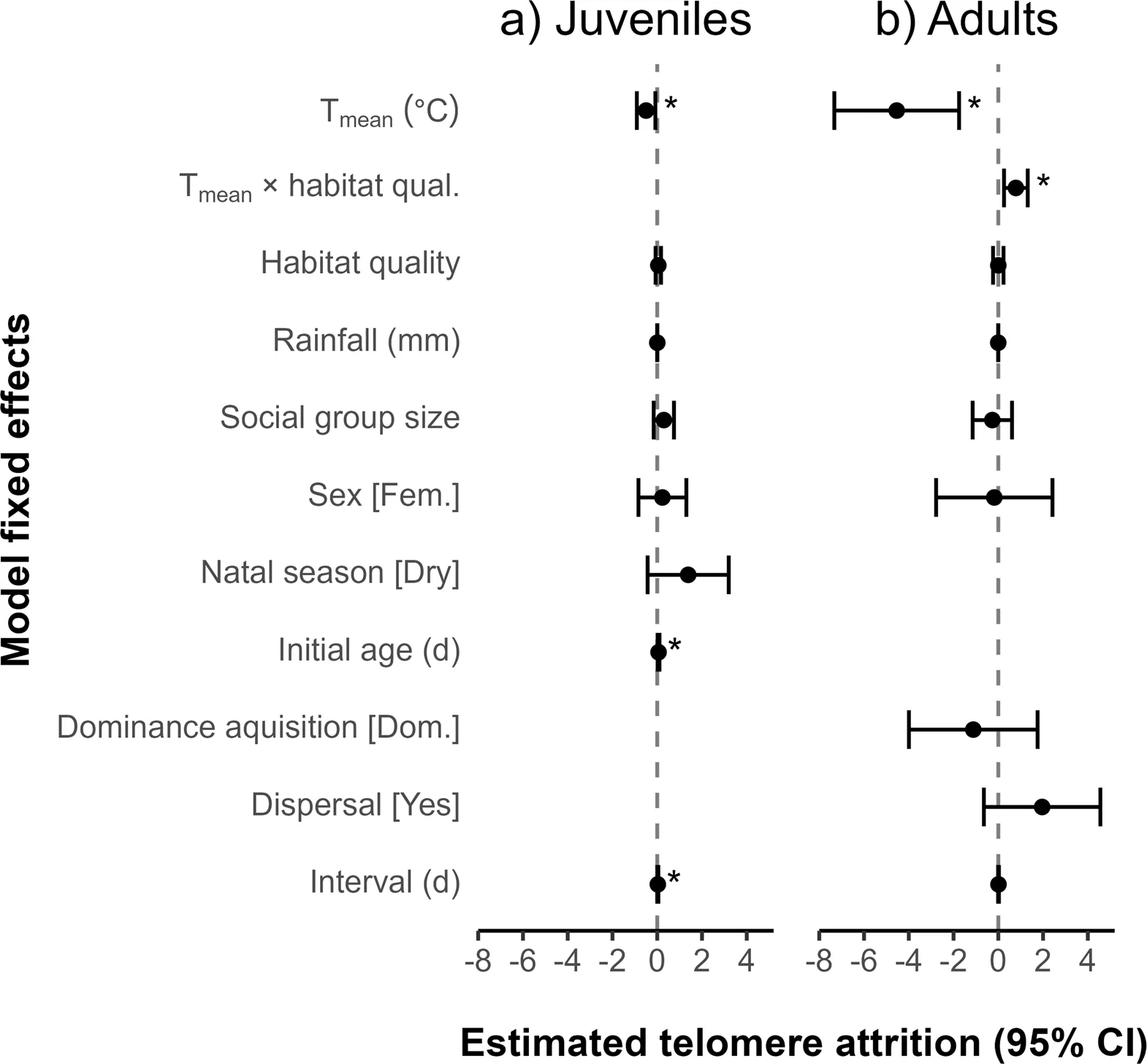
Effect estimates (± s.e.) on telomere attrition in (a) juvenile and (b) adult purple-crowned fairy-wrens. The results indicate a negative and long-term effect of mean daily maximum air temperature (*T*_mean_) on telomere attrition (second telomere measure minus the first, i.e. negative estimates denote attrition). To assess the individual, social and environmental predictors of telomere dynamics, we performed a linear mixed-effects model with restricted maximum likelihood estimation for each life-history stage separately. Potential dispersal and acquisition of dominant breeding position only occur during the transition from subadult to adult. This figure shows the model-derived fixed estimates and 95% confidence intervals for the change in telomere length. An asterisk indicates a significant effect (*p* < 0.05). See supplementary material, table S2, for full model details.

For the juvenile stage, 192 individuals were initially sampled during their dependence as either nestlings (4–14 days old) or fledglings (15–56 days old) and again as independent subadults after a minimum of 90 days (juvenile climate window, figure 1) had passed. Field routines enabled the collection of a second, post-independence, telomere sample during their first year, between 120 and 300 days before adulthood (figure 1). This stage encompasses a period during which individuals depend on parental care, are physiologically immature and transition to independent subadults. For juveniles, each climate variable was calculated between the first capture and 90 days later, corresponding to the approximate start of full independence.

The second life-history stage involved the transition from independent subadult to adulthood (*n* = 77). This stage is characterized by sexual maturity, social transition into a subordinate helper (*n* = 43) or dominant breeder (*n* = 34) and possibly dispersal from their natal territory (25 dispersed, 52 remained on their natal territory). The initial sample included subadults aged between 265 and 365 days, with the second sample approximately 365 days later during the second year of life (365–730 days; figure 1). The climate interval of 365 days later was chosen based on the timing of the fieldwork sampling effort, which corresponds with this critical life stage transition.

### (c) Environmental conditions

We estimated habitat quality based on the volume of *P. aquaticus* in each territory. *Pandanus* is crucial for purple-crowned fairy-wren breeding and has been associated with productivity and individual quality indicators in our study population [35–37,40,41]. Furthermore, it is feasible that the quantity of relatively dense *Pandanus* vegetation may be an important moderator of microclimate [12]. Habitat quality was measured using an index of *Pandanus* density using a well-established protocol [37,40,42], from zero (no *Pandanus*) to 20 (high-quality, dense *Pandanus*). Habitat quality was scored between April–June and September–December, denoting wet and dry season conditions, respectively. For this study, habitat quality scores were obtained nearest to each telomere sampling date and then averaged to determine the mean habitat quality score during the sampling interval (juveniles: mean = 9.67, s.d. = 4.98, range = 0–19.30 unit scores; adults: mean = 10.96, s.d. = 4.72, range = 1.64–18.73 unit scores).

**Figure 3.**
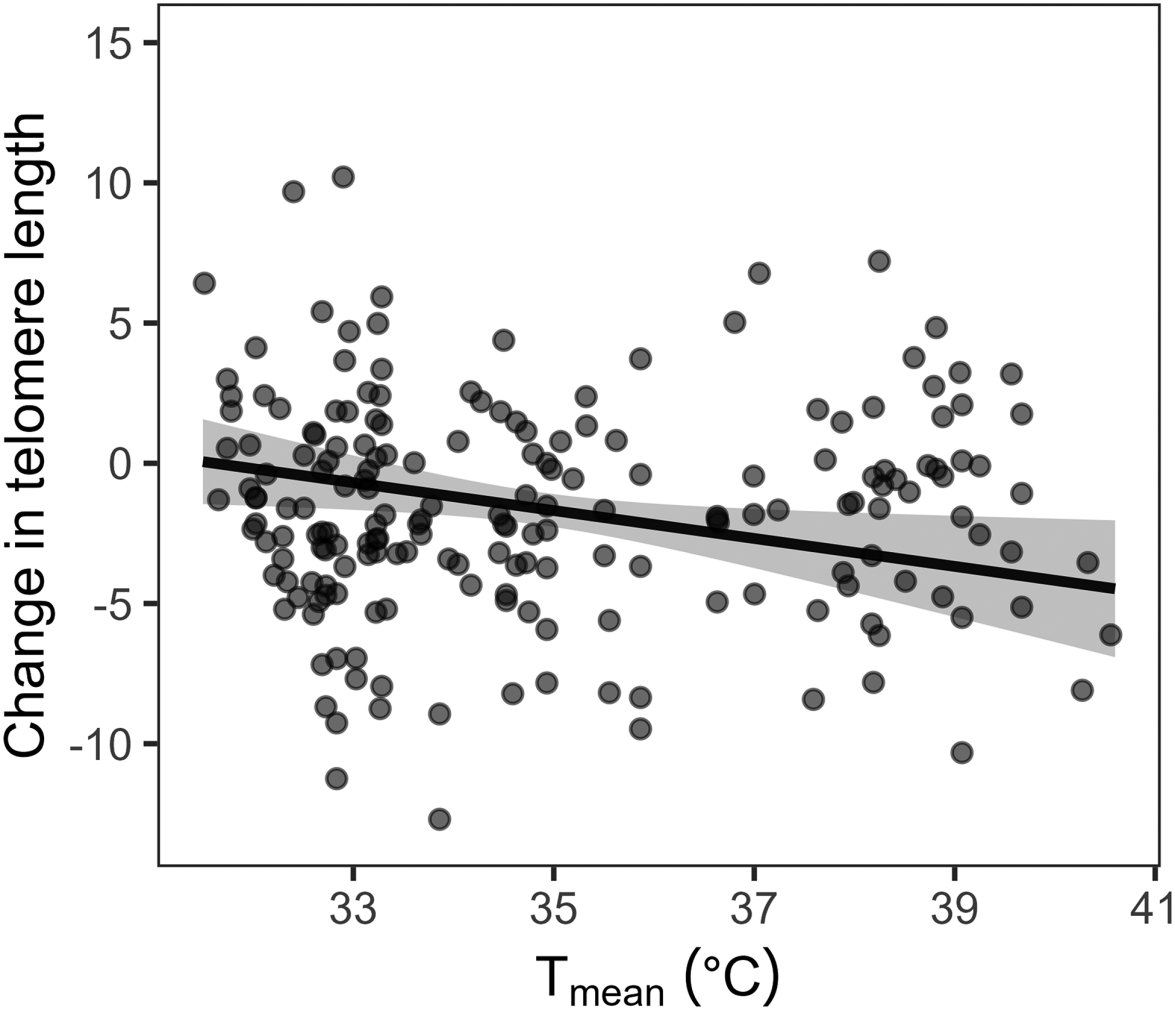
Hotter mean air temperature is associated with increased juvenile telomere attrition (*n* = 192) in purple-crowned fairy-wrens. Individual telomere change data points were calculated by subtracting the initial length from the second (negative values indicate telomere attrition). Regression lines and confidence intervals were derived from model estimates.

Hot (mean maximum daily air temperature) and dry (rainfall and season) conditions are critical predictors of nestling telomere length [26]. Therefore, we aimed to incorporate a set of variables that assessed these conditions during the climate windows of interest (i.e. juvenile vs. adult stages, figure 1). We obtained daily maximum air temperature data from two nearby Australian Bureau of Meteorology weather stations, Mornington station (site number 002076, 17.51° S, 126.11° E) and Fitzroy Crossing Aero Station (site number 003093, 18.18° S, 125.56° E, located approximately 93 km from the field site). Following [26], we performed a linear regression model between maximum daily temperature measures from both weather stations and used the slope and intercept to interpolate missing values for our Mornington field site, a metric highly correlated with nest microclimate temperature in this species [26]. Rainfall data were available from the Mornington weather station. For this study, there were two climate windows of biological interest spanning distinct life-history stages (figure 1; see §2(e)). We calculated the same climate variables for both the 90-day juvenile and 365-day adult windows. These included mean daily maximum air temperature (*T*_mean_), mean daily temperature range (daily maximum minus minimum °C; *T*_range_) and the frequency of days over 40°C (*T*_40_). The latter two variables were included to test for the association between climate variability and extreme temperatures. However, they were not included in the model concurrently owing to the high correlation with mean temperature (supplementary material, figure S1) and were not the focus of this study. Minimum daily environmental temperature did not affect telomere length in purple-crowned fairy-wren nestlings [26] and, therefore, was not included in this study. Owing to the significance of rainfall in the life cycle of purple-crowned fairy-wrens [31], its potential to mitigate the effects of extreme heat [26] and known effects in other species [43], we included total rainfall (mm) during each period to assess the impact of water availability in the system.

### (d) Relative telomere length quantification

We used an automated QIAamp DNA kit (Qiagen) method using a QIAcube HT instrument to obtain high-quality DNA for telomere quantification [38]. DNA purity and concentration were assessed using a NanoDrop (ND-1000), and DNA integrity was assessed on an agarose gel for a smear indicating degradation [38]. Acceptable quality control measures were identical to those previously reported and shown to produce reliable telomere estimates [38].

To measure relative telomere length, we used a quantitative real-time polymerase chain reaction (qPCR) method [44], validated for use in purple-crowned fairy-wrens [38]. In brief, qPCR reactions were prepared by automation in an EpMotion 5075 into 96-well plates. The total reaction volume of 25 µl included 12.5 µl of SYBR Green I (Roche), 300 nM of each normalizing control gene (glyceraldehyde-3-phosphate dehydrogenase; GAPDH) primer [45] or 400 nM of each telomere primer (Integrated DNA Technologies) and 10 ng of sample DNA (except the twofold serial dilution containing 40, 20, 10, 5 and 2.5 ng of DNA). All reactions were added in duplicate except for the inter-plate control, which was included in four separate wells. The telomere primers were Tel1b 5′ - CGG TTT GTT TGG GTT TGG GTT TGG GTT TGG GTT TGG GTT - 3′ and Tel2b 5′ - GGC TTG CCT TAC CCT TAC CCT TAC CCT TAC CCT TAC CCT - 3′ [44]. The GAPDH primers were GT2-GAPDH-forward 5′ - CCA TCA CAG CCA CAC AGA AG - 3′ and GT2-GAPDH-reverse 5′ - TTT TCC CAC AGC CTT AGC AG - 3′ [45]. Reactions were run on a LightCycler 480 (Roche) machine on separate plates as follows: telomere (95°C for 15 min, followed by 35 cycles of 15 s at 95°C, 30 s at 56°C, 30 s at 72°C) and GAPDH (95°C for 15 min, followed by 40 cycles of 15 s at 95°C, 30 s at 60°C, 30 s at 72°C). A melt-curve analysis followed both reactions to ensure the correct product was amplified. Efficiencies (90%–99% for telomere and GAPDH, respectively) and intra- and inter-assay repeatabilities were high and have been previously reported [38]. The calculation of relative telomere length was identical to the methods previously described [38], but was adjusted for potential sources of error as described in [46], see Materials and methods section in the supplementary material.

### (e) Statistical analysis

All analyses were performed using the statistical software R version 4.3.1 [47]. We tested whether telomere dynamics were associated with the environmental or social context using a linear mixed-effects model with restricted maximum likelihood estimation, using the *lme4* and *lmerTest* packages [48]. We fitted a global model for each stage with the change in telomere length (second telomere measure minus initial telomere measure) as the dependent variable. Thus, negative values indicate telomere attrition and positive values indicate lengthening.

First, for the juvenile model (*n* = 192 individuals, figure 1), we included mother ID, territory ID and natal year (austral calendar, July–June) as random terms to control for sibship, spatial and cohort non-independence. However, territory and natal year were excluded because they explained zero variance. Sex was included as a fixed factor to control for sex-dependent differences in telomere attrition [26]. Telomere attrition occurs throughout life [21] and is greatest earlier in life in this species [49]. Therefore, we controlled for the age at initial sampling (days), sampling time interval (days) and their interaction. The latter was included because telomere loss is partly a function of cell renewal and growth, which vary with time. We included group size as a covariate for the social environment and an approximation of bird density. Group size was defined in this analysis as the mean number of independent birds on the territory, calculated from the number of independent birds (greater than 120 days of age) on the territory at each telomere sampling date. For habitat quality, we included mean habitat quality as a fixed covariate (defined above) and natal season (wet or dry) as a measure of seasonal variation not detected by climate variables. For climate variables, we included juvenile *T*_mean_ and total rainfall (mm) as fixed covariates. To determine whether rainfall, habitat quality and social environment (group size) could buffer suboptimal thermal conditions, we included three two-way interactions with environmental temperature.

Second, we fitted a model to investigate adult telomere dynamics, measured as the change in telomere length during the first year of adulthood (*n* = 77 individuals, figure 1). The adult-stage model was similar to the juvenile model. However, we excluded age first captured, as all individuals were caught in their first year at the same life stage when telomere attrition is relatively stable [49]. Year of the initial capture (austral; July–June) was included as a random term to control for unmeasured environmental variation, but was subsequently removed because it explained less than 0.001% of the variance and did not qualitatively change the results. Unique to the adult stage, individuals fall into two distinct social classes (dominant breeder or subordinate helper) and may disperse from their natal territory. Therefore, we included two additional factors to test whether this life-history period of change impacts telomere dynamics, social status (dominant/subordinate) and natal dispersal (dispersed or remained). To explore the possibility that temperature extremes or variability better explained any outcomes compared to mean temperatures, we substituted *T*_mean_ with either *T*_40_ or *T*_range_ into the final models. Models with *T*_mean_, *T*_range_ and *T*_40_ were compared within each life-history stage using corrected Akaike information criterion (AIC_c_) values. However, in both juveniles and adults, we found no evidence to suggest that temperature extremes or variability outperformed mean temperature conditions (supplementary material, tables S2 and S3, figure S2).

In all models, we sequentially removed non-significant interactions (*p* ≥ 0.05) from the final model while retaining all main effects. All covariates were mean-centred, and collinearity was checked between all variables using variance inflation factors (VIFs), which were all within acceptable limits (VIF ≤ 3.7). Models were checked to ensure that the assumptions were not violated. All models are reported in the supplementary material.

## 3. Results and discussion

### (a) Heat exposure increases telomere attrition during the juvenile life stage

To investigate how environmental temperature affects telomere maintenance during the juvenile life-history stage, we measured telomere length during the dependent stage when birds are nutritionally dependent on their parents (nestling or fledgling, less than or equal to 56 days old) and again after a 90-day climate window (figure 1) that coincides with their transition to nutritional independence (termed ‘juvenile stage’; see Material and methods). Owing to our large, high-quality dataset, the analyses could control for many individual, social and ecological factors that may potentially confound the effect of climate on telomere attrition (whereby only age and sampling interval duration were significant; figure 2 and supplementary material, figure S3). Our analyses show that juveniles experience increased telomere attrition when exposed to hotter environmental conditions during this period (figures 2 and 3; supplementary material, table S4). The thermal conditions during the juvenile climate window were typical of a tropical climate, with a mean daily maximum air temperature (*T*_mean_) of 34.9°C (range: 31.6°C–40.6°C). In the upper half of the range, juveniles are likely to be above their thermoneutral zone [50,51] and required to engage in behavioural and/or physiological thermoregulatory responses, potentially reaching or exceeding their heat tolerance limit. Concomitant decreases in resource availability, increased metabolic by-products or oxidative damage can have long-term costs by reducing the somatic state, as expressed in the acceleration of telomere attrition [20]. Instead of, or in addition to, a direct physiological mechanism hypothesis, it is also possible that environmental temperatures correlate with seasonal variation in other factors (e.g. lower arthropod (food) availability [31,52]) that accelerate telomere attrition. However, this alternative hypothesis may be less likely because temperature effects were independent of natal season (tropical wet-dry climate; see Material and methods; figure 2). Thus, sublethal hot conditions are likely to be physiologically challenging for juveniles and may compel individuals to expend resources on thermoregulation for immediate survival, at the cost of somatic maintenance and long-term survival. Although a strength of our study is that individuals are followed across different life-history phases under heterogeneous natural conditions, it is exclusively observational and cannot demonstrate causality. Experimental manipulations on this temporal and spatial scale appear unfeasible, although greater insights into the drivers of telomere dynamics could be derived from investigations into physiological mechanisms or short-term experiments.

**Figure 4.**
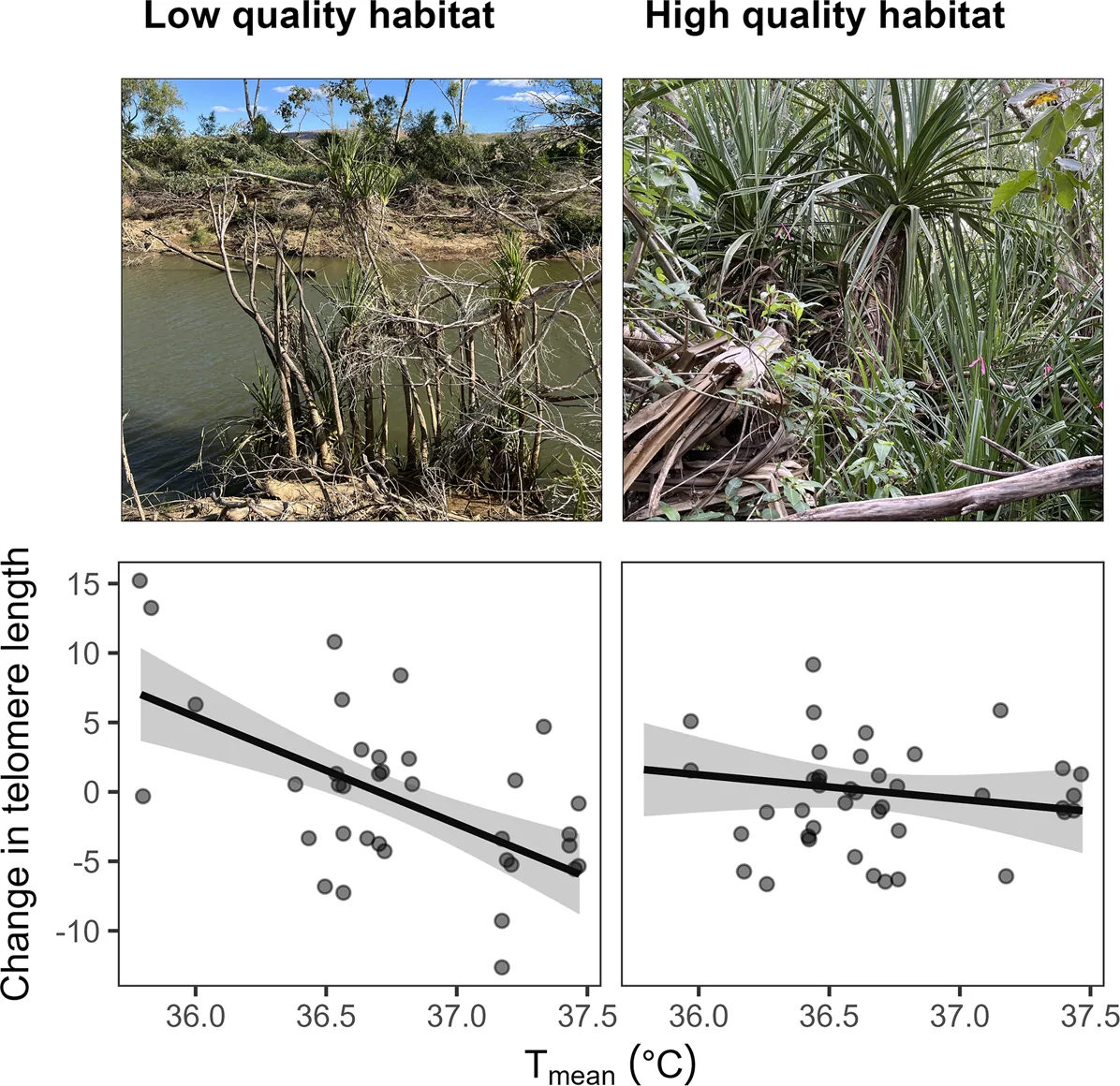
Adult purple-crowned fairy-wrens are more susceptible to hotter conditions when they live in lower quality habitat (*n* = 77). Mean daily maximum air temperature is negatively related to telomere length change (negative values denote telomere shortening) in low-quality habitat with low vegetation cover (left). However, this pattern is weaker in high-quality habitat with denser *Pandanus* vegetation (*right*). To visualize the temperature × habitat quality interaction, we partitioned our raw data into two categories: below- and above-mean habitat quality values (habitat quality was calculated as a weighted average of habitat quality during the sampling interval between a 0–20 scale: mean = 11, s.d. = 4.72, range = 1.64–18.73: note that habitat quality was modelled as a continuous variable). We then plotted model-derived regression lines and confidence intervals for the temperature-telomere effect based on the mean habitat quality score within each category (solid lines). As a reference, the images show low- and high-quality habitat with scores of 2 and 17, respectively. Mean daily maximum air temperature during the adult stage ranged between 35.8°C and 37.4°C (mean = 36.7°C, s.d. = 0.4). Photos: Ariana M La Porte and AWC.

We found no evidence that ecological, climatic or social conditions mitigated the effect of heat exposure on telomere loss. Although water availability plausibly buffers thermoregulatory challenges [5,12,53], rainfall did not buffer the impact of high *T*_mean_ on telomere attrition in juveniles (supplementary material, table S5), nor was there an independent effect of rainfall on telomere attrition (supplementary material, table S4 and figure S5). As higher rainfall and wet season conditions mitigated the negative effect of heat exposure on telomere length in 7-day-old purple-crowned fairy-wren nestlings [26], we predicted a similar positive effect on telomere attrition in juveniles. A lack of positive rainfall effects is thus surprising and contrary to our predictions. Similarly, we predicted positive effects of habitat quality, measured as the density of *P. aquaticus* riparian vegetation, essential habitat for the (highly territorial) species [54,55]. However, the quality of the habitat experienced over the juvenile stage was not related to telomere attrition (figure 2) and did not buffer the effect of temperature (supplementary material, table S5). The absence of evidence for a buffering effect of habitat quality on the effect of temperature was also unexpected, as denser riparian habitat is generally cooler, and *Pandanus* density predicts arthropod prey biomass, water availability during dry periods [42], social group size [56], reproductive success [57] and breeder settlement decisions [40].

The lack of any evidence for a benefit from larger social group size on thermal impacts was less unexpected. As purple-crowned fairy-wrens are cooperative breeders, juveniles remain dependent on group members for food for approximately three months. This could help juveniles cope with suboptimal conditions as they can receive additional care when needed. However, larger group sizes are not only beneficial, as all group members also compete for the limited resources available in the territory. Cooperative behaviour in purple-crowned fairy-wrens is carefully calibrated to costs and benefits: group members may reduce or refrain from contributing [34,58], or be antagonistic or compete, depending on context [37,59]; for example, larger social groups are associated with shorter nestling telomeres [26]. Possibly, this balance between the benefits of cooperation and the costs of group living explains why group size does not affect telomere attrition rates or the impact of hot conditions in juveniles (figure 2; supplementary material, table S5).

### (b) High-quality habitat mitigates heat-associated telomere attrition in adults

Given that adult purple-crowned fairy-wrens have a lower mortality rate [60] and a slower rate of telomere attrition [24], we hypothesized that adults would be less susceptible to high temperatures than juveniles or nestlings [12,61]. To investigate temperature effects on telomere dynamics in adults, we measured telomere length in individuals first when they were independent subadults and again after a 365-day climate window (termed ‘adult stage’; figure 1; see Material and methods). This period is characterized by the first moult into sexually dimorphic adult plumage, and the potential acquisition of a breeding position through dispersal or inheritance [40,41,56,62]. By contrast to our expectation, we found that *T*_mean_ affected telomere dynamics during the adult stage, no less strongly than during the juvenile phase (figure 2; supplementary material, table S4). However, the strength of the association depended on the quality of the habitat the individual was living in (figures 2 and 4; see Material and methods). For adults inhabiting low-quality habitat, mean maximum temperature was strongly negatively associated with telomere attrition (figure 4; supplementary material, figure S5), while the negative impact of temperature was increasingly weaker as habitat quality increased. Low-quality habitats, characterized by less dense *Pandanus* (mid-storey) vegetation, will probably be hotter because of higher rates of exposure to solar radiation, while high-density *Pandanus* territories are cooler owing to increased shade [14], but direct measurements are not currently available. However, habitat quality is also associated with social behaviour (mate retention [40], aggressiveness [62]) and lower quality habitat supports less prey biomass, smaller social groups and lower productivity [32,35,40], suggesting reduced resources for somatic maintenance. Therefore, the increased attrition of telomeres in response to high mean temperature in poor-quality habitat may be attributed to a suite of habitat characteristics in addition to the direct effects of heat. Furthermore, habitat occupancy may be correlated with individual condition, which may lead to the observation that individuals in better condition can cope with hotter environments and vice versa. Therefore, we tested whether habitat quality at first capture was associated with initial telomere length while controlling for age, sex, body condition (mass and tarsus) and season (wet/dry). Contrary to the expectation that individuals in better condition preferentially occupy higher quality territories, we found that initial telomere length was negatively associated with habitat quality (*β* = −0.12 ± 0.05 s.e., *p* = 0.02; supplementary material, table S6). Thus, reduced telomere shortening observed in high-quality territories during hot conditions is unlikely to be explained solely by pre-existing differences in telomere length among individuals occupying different territories. Regardless of the mechanism, our results demonstrate that adult birds exposed to high temperatures are better off in intact, dense vegetation.

Besides the climate and habitat interaction, and similar to juveniles, we found no evidence to suggest other individual (e.g. sex) or social covariates to predict telomere attrition in adults (figure 2; supplementary material, table S4). Adults differ from juveniles in their potential for dispersal and their roles as subordinate helpers or dominant breeders, but whether or not birds transitioned from subadult to a subordinate or dominant adult or dispersed during the sampling interval did not predict telomere attrition (figure 2; supplementary material, table S4; note that our study design allows us to distinguish dispersal from death with high probability). The absence of biological factors or life events affecting telomere attrition across two distinct and important life-history stages suggests that life-history trade-offs are being adjusted or individually optimized according to resource availability and/or individual heterogeneity [63,64]. This puts into sharp relief the impact of hot temperatures across both life stages.

### (c) Heat exposure increases telomere loss throughout life—implications in a warming world

A variety of adverse conditions are known to negatively impact telomere dynamics during early life [15], when sensitivity to environmental and climatic stressors [65], as well as telomere attrition, tend to be greatest [49]. Previously, we found in purple-crowned fairy-wrens that hot and dry conditions during the nestling stage result in shorter nestling telomeres [26], and others have shown complex patterns between telomere length and heat in young in other vertebrates [28,29,66–74]. Here, we show that these impacts of heat exposure on telomere dynamics extend years beyond the nestling stage, well into adulthood (figure 2). Interestingly, and against expectations, the effect of temperature on telomere attrition was substantially larger in adults than in juveniles, overall (figure 2) and particularly in lower quality habitat (figure 2). A likely explanation for the unexpected lower sensitivity of the younger life-history stage is the slow pace of life in this species: individuals continue to be cared for by other adults throughout the juvenile stage [31], and the acquisition of nutritional independence requires several months [31]. By contrast, adults are not only self-reliant but are also required to start looking and competing for breeding opportunities, either at home or elsewhere. This may increase the likelihood of heat exposure or limit resources available for self-maintenance and thermoregulation. In this regard, the absence of any significant effect of dominance acquisition or dispersal on telomere dynamics is consistent with all subadults attempting these activities, the costs of which might be equivalent regardless of success or failure. Ultimately, all life stages of purple-crowned fairy-wrens—from nestlings [26] to adults (this study)—were affected by hot thermal conditions, especially those without access to very dense vegetation.

The negative impact of hotter temperatures on somatic maintenance, regardless of the underlying mechanism, raises concerns about the future viability of populations in a warming world. In many species, including purple-crowned fairy-wrens, telomere dynamics predict lifespan [16,24] and lifetime reproductive success [60]. Therefore, as global temperatures continue to rise [1,2], a hidden reduction in fitness could accumulate, potentially leading to population decline [6,26,68]. Large historical reductions in the abundance of tropical birds, in particular, are attributable to past increases in hot temperatures owing to global warming [8,9], and our data suggest that such declines potentially relate to the impacts of sublethal temperature [10,75,76]. This is particularly true if species are contending with additional threats, such as habitat destruction, to which tropical savannahs are particularly vulnerable [77]. Purple-crowned fairy-wrens are an indicator species for the health and conservation management of their riparian ecosystem [55], which is an important refuge for climate-sensitive birds of the region [78,79]. Despite the negative outlook for climate warming, our findings demonstrate that high-quality habitat can reduce the effect of high temperature (figure 4; supplementary material, figure S5). Thus, protecting riparian vegetation from sources of degradation, including high-intensity fire [80] and grazing [55,81], can reduce the impact of climate warming on endangered purple-crowned fairy-wrens and species that inhabit riparian zones [78,82]. Overall, this study also highlights the danger of sublethal temperatures and the value of conserving climate refugia as a management strategy in protecting species from the cryptic effects of future climate warming.

## Supplementary Material



## Data Availability

All data used in this study are available on Dryad [83]. Supplementary material is available online [84].

## References

[1] Meehl GA , Tebaldi C. 2004 More intense, more frequent, and longer lasting heat waves in the 21st century. Science 305, 994–997. (doi:10.1126/science.1098704) 15310900

[2] Murali G , Iwamura T, Meiri S, Roll U. 2023 Future temperature extremes threaten land vertebrates. Nature 615, 461–467. (doi:10.1038/s41586-022-05606-z) 36653454

[3] Halupka L . 2023 The effect of climate change on avian offspring production: a global meta-analysis. Proc. Natl Acad. Sci. USA 120, e2208389120. (doi:10.1073/pnas.2208389120) PMC10175715 37126701

[4] Conradie SR , Woodborne SM, Cunningham SJ, McKechnie AE. 2019 Chronic, sublethal effects of high temperatures will cause severe declines in southern African arid-zone birds during the 21st century. Proc. Natl Acad. Sci. USA 116, 14 065–14 070. (doi:10.1073/pnas.1821312116) PMC6628835 31235571

[5] Albright TP , Mutiibwa D, Gerson AR, Smith EK, Talbot WA, O'Neill JJ, McKechnie AE, Wolf BO. 2017 Mapping evaporative water loss in desert passerines reveals an expanding threat of lethal dehydration. Proc. Natl Acad. Sci. USA 114, 2283–2288. (doi:10.1073/pnas.1613625114) PMC5338552 28193891

[6] León GM-D , Thakur MP. 2024 Ecological debts induced by heat extremes. Trends Ecol. Evol. 39, 1024–1034. (doi:10.1016/j.tree.2024.07.002) 39079760

[7] McKechnie AE , Wolf BO. 2009 Climate change increases the likelihood of catastrophic avian mortality events during extreme heat waves. Biol. Lett. 6, 253–256. (doi:10.1098/rsbl.2009.0702) PMC2865035 19793742

[8] Wiens JJ . 2016 Climate-related local extinctions are already widespread among plant and animal species. PLoS Biol. 14, e2001104. (doi:10.1371/journal.pbio.2001104) PMC5147797 27930674

[9] Kotz M , Amano T, Watson JEM. 2025 Large reductions in tropical bird abundance attributable to heat extreme intensification. Nat. Ecol. Evol. 9, 1897–1909. (doi:10.1038/s41559-025-02811-7) 40790263

[10] Sherry TW . 2021 Sensitivity of tropical insectivorous birds to the anthropocene: a review of multiple mechanisms and conservation implications. Front. Ecol. Evol. 9, 1897–1909. (doi:10.3389/fevo.2021.662873)

[11] Schou MF , Cornwallis CK. 2024 Adaptation to fluctuating temperatures across life stages in endotherms. Trends Ecol. Evol. 39, 841–850. (doi:10.1016/j.tree.2024.05.012) 38902165

[12] Cunningham SJ , Gardner JL, Martin RO. 2021 Opportunity costs and the response of birds and mammals to climate warming. Front. Ecol. Environ. 19, 300–307. (doi:10.1002/fee.2324)

[13] Scheffers BR , Edwards DP, Diesmos A, Williams SE, Evans TA. 2014 Microhabitats reduce animal's exposure to climate extremes. Glob. Change Biol. 20, 495–503. (doi:10.1111/gcb.12439) 24132984

[14] De Frenne P , Zellweger F, Rodríguez-Sánchez F, Scheffers BR, Hylander K, Luoto M, Vellend M, Verheyen K, Lenoir J. 2019 Global buffering of temperatures under forest canopies. Nat. Ecol. Evol. 3, 744–749. (doi:10.1038/s41559-019-0842-1) 30936433

[15] Chatelain M , Drobniak SM, Szulkin M. 2019 The association between stressors and telomeres in non-human vertebrates: a meta-analysis. Ecol. Lett. 23, 381–398. (doi:10.1111/ele.13426) 31773847

[16] Eastwood JR , Dupoué A, Delhey K, Verhulst S, Cockburn A, Peters A. 2023 When does early-life telomere length predict survival? A case study and meta-analysis. Mol. Ecol. 32, 3000–3013. (doi:10.1111/mec.16894) 36811398

[17] Wilbourn RV , Moatt JP, Froy H, Walling CA, Nussey DH, Boonekamp JJ. 2018 The relationship between telomere length and mortality risk in non-model vertebrate systems: a meta-analysis. Phil. Trans. R. Soc. B 373, 20 160 447. (doi:10.1098/rstb.2016.0447) PMC5784067 29335371

[18] Blackbum EH . 1991 Telomeres. Trends Biochem. Sci. 16, 378–381. (doi:10.1016/0968-0004(91)90155-O) 1785140

[19] Shay JW , Wright WE. 2019 Telomeres and telomerase: three decades of progress. Nat. Rev. Genet. 20, 299–309. (doi:10.1038/s41576-019-0099-1) 30760854

[20] Armstrong E , Boonekamp J. 2023 Does oxidative stress shorten telomeres *in vivo*? A meta-analysis. Ageing Res. Rev. 85, 101 854. (doi:10.1016/j.arr.2023.101854) 36657619

[21] Remot F , Ronget V, Froy H, Rey B, Gaillard J-M, Nussey DH, Lemaitre J-F. 2022 Decline in telomere length with increasing age across nonhuman vertebrates: a meta-analysis. Mol. Ecol. 31, 5917–5932. (doi:10.1111/mec.16145) 34437736

[22] Badás EP , Bauch C, Boonekamp JJ, Mulder E, Verhulst S. 2023 Ectoparasite presence and brood size manipulation interact to accelerate telomere shortening in nestling jackdaws. Mol. Ecol. 32, 6913–6923. (doi:10.1111/mec.17177) 37864481

[23] Monaghan P , Ozanne SE. 2018 Somatic growth and telomere dynamics in vertebrates: relationships, mechanisms and consequences. Phil. Trans. R. Soc. B 373, 20 160 446. (doi:10.1098/rstb.2016.0446) PMC5784066 29335370

[24] Sheldon EL *et al.* 2022 Telomere dynamics in the first year of life, but not later in life, predict lifespan in a wild bird. Mol. Ecol. 31, 6008–6017. (doi:10.1111/mec.16296) 34850488

[25] Furic C , Marciau C, Hsu B-Y, Cossin-Sevrin N, Fleitz J, Reichert S, Ruuskanen S, Stier A. 2026 Warm and cool temperatures decrease early-life telomere length in wild pied flycatchers. J. Avian Biol. 2026, 03 511. (doi:10.1002/jav.03511)

[26] Eastwood JR , Connallon T, Delhey K, Hall ML, Teunissen N, Kingma SA, La Porte AM, Verhulst S, Peters A. 2022 Hot and dry conditions predict shorter nestling telomeres in an endangered songbird: implications for population persistence. Proc. Natl Acad. Sci. USA 119, e2122944119. (doi:10.1073/pnas.2122944119) PMC9231487 35696588

[27] Eastwood JR , Dupoué A, Verhulst S, Cockburn A, Peters A. 2023 Cool, dry nights and short heatwaves during growth result in longer telomeres in temperate songbird nestlings. Mol. Ecol. 32, 5382–5393. (doi:10.1111/mec.17107) 37606092

[28] Pepke ML , Kvalnes T, Ranke PS, Araya-Ajoy YG, Wright J, Sæther B-E, Jensen H, Ringsby TH. 2022 Causes and consequences of variation in early-life telomere length in a bird metapopulation. Ecol. Evol. 12, e9144. (doi:10.1002/ece3.9144) PMC9339764 35923948

[29] van Lieshout S , Badás EP, Bright Ross JG, Bretman A, Newman C, Buesching CD, Burke T, Macdonald DW, Dugdale H. 2022 Early-life seasonal, weather and social effects on telomere length in a wild mammal. Mol. Ecol. 31, 5993–6007. (doi:10.1111/mec.16014) 34101279

[30] Skroblin A , Teunissen T, Peters A, Greatwich B, Legge SM, van Doorn A, Burbidge AH, Garnett ST. 2021Western purple-crowned fairy-wren *Malurus coronatus coronatus*. In The action plan for Australian birds 2020 (eds Garnett ST, Baker GB), pp. 501–504. Melbourne, Australia: CSIRO Publishing.

[31] Hidalgo Aranzamendi NH , Hall ML, Kingma SA, van de Pol M, Peters A. 2019 Rapid plastic breeding response to rain matches peak prey abundance in a tropical savanna bird. J. Anim. Ecol. 88, 1799–1811. (doi:10.1111/1365-2656.13068) 31407349

[32] Kingma SA , Hall ML, Peters A. 2011 No evidence for offspring sex-ratio adjustment to social or environmental conditions in cooperatively breeding purple-crowned fairy-wrens. Behav. Ecol. Sociobiol. 65, 1203–1213. (doi:10.1007/s00265-010-1133-7)

[33] Hall ML , Peters A. 2008 Coordination between the sexes for territorial defence in a duetting fairy-wren. Anim. Behav. 76, 65–73. (doi:10.1016/j.anbehav.2008.01.010)

[34] Teunissen N , Fan M, Roast MJ, Hidalgo Aranzamendi N, Kingma SA, Peters A. 2023 Best of both worlds? Helpers in a cooperative fairy-wren assist most to breeding pairs that comprise a potential mate and a relative. R. Soc. Open Sci. 10, 231342. (doi:10.1098/rsos.231342) PMC10646452 38026024

[35] Kingma SA , Hall ML, Arriero E, Peters A. 2010 Multiple benefits of cooperative breeding in purple-crowned fairy-wrens: a consequence of fidelity? J. Anim. Ecol. 79, 757–768. (doi:10.1111/j.1365-2656.2010.01697.x) 20443991

[36] Fan M , Hall ML, Kingma SA, Mandeltort LM, Hidalgo Aranzamendi N, Delhey K, Peters A. 2017 No fitness benefits of early molt in a fairy-wren: relaxed sexual selection under genetic monogamy? Behav. Ecol. 28, 1055–1067. (doi:10.1093/beheco/arx065)

[37] Teunissen N , Kingma SA, Peters A. 2025 Climate, habitat and demography predict dispersal by an endangered bird in a fragmented landscape. J. Appl. Ecol. 62, 1127–1136. (doi:10.1111/1365-2664.70026)

[38] Eastwood JR , Mulder E, Verhulst S, Peters A. 2018 Increasing the accuracy and precision of relative telomere length estimates by RT qPCR. Mol. Ecol. Resour. 18, 68–78. (doi:10.1111/1755-0998.12711) 28805012

[39] Griffiths R , Double MC, Orr K, Dawson RJG. 1998 A DNA test to sex most birds. Mol. Ecol. 7, 1071–1075. (doi:10.1046/j.1365-294x.1998.00389.x) 9711866

[40] Hidalgo Aranzamendi N , Hall ML, Kingma SA, Sunnucks P, Peters A. 2016 Incest avoidance, extrapair paternity, and territory quality drive divorce in a year-round territorial bird. Behav. Ecol. 27, 1808–1819. (doi:10.1093/beheco/arw101)

[41] Nolazco S , Hall ML, Kingma SA, Delhey K, Peters A. 2020 No evidence for an adaptive role of early molt into breeding plumage in a female fairy wren. Behav. Ecol. 31, 411–420. (doi:10.1093/beheco/arz203)

[42] Roast MJ , Aulsebrook AE, Fan M, Hidalgo Aranzamendi N, Teunissen N, Peters A. 2019 Short-term climate variation drives baseline innate immune function and stress in a tropical bird: a reactive scope perspective. Physiol. Biochem. Zool. 92, 140–151. (doi:10.1086/702310) 30689489

[43] Wood EM , Capilla-Lasheras P, Cram DL, Walker LA, York JE, Lange A, Hamilton PB, Tyler CR, Young AJ. 2022 Social dominance and rainfall predict telomere dynamics in a cooperative arid-zone bird. Mol. Ecol. 31, 6141–6154. (doi:10.1111/mec.15868) 33657651

[44] Criscuolo F , Bize P, Nasir L, Metcalfe NB, Foote CG, Griffiths K, Gault EA, Monaghan P. 2009 Real-time quantitative PCR assay for measurement of avian telomeres. J. Avian Biol. 40, 342–347. (doi:10.1111/j.1600-048X.2008.04623.x)

[45] Atema E , van Oers K, Verhulst S. 2013 GAPDH as a control gene to estimate genome copy number in great tits, with cross-amplification in blue tits. Ardea 101, 49–54. (doi:10.5253/078.101.0107)

[46] Evans J , Eastwood J, Teunissen N, Verhulst S, Mikheyev A, Peters A. 2026 Mothers matter most: maternal, but not paternal, age and inbreeding affect nestling telomere length in a wild passerine. Mol. Ecol. 35, e70437. (doi:10.1111/mec.70437) PMC13270259 42299488

[47] R Core Team . 2023 R: a language and environment for statistical computing. Vienna, Austria: R Foundation for Statistical Computing. https://www.R-project.org/.

[48] Bates D , Mächler M, Bolker B, Walker S. 2015 Fitting linear mixed-effects models using lme4. J. Stat. Softw. 1, 1–48. (doi:10.18637/jss.v067.i01)

[49] Roast MJ , Eastwood JR, Aranzamendi NH, Fan M, Teunissen N, Verhulst S, Peters A. 2022 Telomere length declines with age, but relates to immune function independent of age in a wild passerine. R. Soc. Open Sci. 9, 212 012. (doi:10.1098/rsos.212012) PMC9043702 35601455

[50] Pollock HS , Brawn JD, Cheviron ZA. 2021 Heat tolerances of temperate and tropical birds and their implications for susceptibility to climate warming. Funct. Ecol. 35, 93–104. (doi:10.1111/1365-2435.13693)

[51] Lill A , Box J, Baldwin J. 2006 Do metabolism and contour plumage insulation vary in response to seasonal energy bottlenecks in superb fairy-wrens? Aust. J. Zool. 54, 23–30. (doi:10.1071/ZO05029)

[52] Spurgin LG , Bebbington K, Fairfield EA, Hammers M, Komdeur J, Burke T, Dugdale HL, Richardson DS. 2017 Spatio-temporal variation in lifelong telomere dynamics in a long-term ecological study. J. Anim. Ecol. 87, 187–198. (doi:10.1111/1365-2656.12741) PMC5765431 28796902

[53] Bourne AR , Ridley AR, McKechnie AE, Spottiswoode CN, Cunningham SJ. 2021 Dehydration risk is associated with reduced nest attendance and hatching success in a cooperatively breeding bird, the southern pied babbler *Turdoides bicolor*. Conserv. Physiol. 9, coab043. (doi:10.1093/conphys/coab043) PMC8208672 34150211

[54] Rowley I , Russell E. 1993 The purple-crowned fairy-wren *Malurus coronatus*. II. Breeding biology, social organisation, demography and management. Emu 93, 235–250. (doi:10.1071/MU9930235)

[55] Skroblin A , Legge S. 2012 Influence of fine-scale habitat requirements and riparian degradation on the distribution of the purple-crowned fairy-wren (*Malurus coronatus coronatus*) in northern Australia. Austral. Ecol. 37, 874–884. (doi:10.1111/j.1442-9993.2011.02331.x)

[56] Kingma SA , Hall ML, Peters A. 2011 Multiple benefits drive helping behavior in a cooperatively breeding bird: an integrated analysis. Am. Nat. 177, 486–495. (doi:10.1086/658989) 21460570

[57] Teunissen N , Peters A. 2022 Predator suppression by a toxic invader does not cascade to prey due to predation by alternate predators. Biol. Invasions 24, 2723–2733. (doi:10.1007/s10530-022-02808-4)

[58] Teunissen N , Kingma SA, Fan M, Roast MJ, Peters A. 2021 Context-dependent social benefits drive cooperative predator defense in a bird. Curr. Biol. 31, 4120–4126.e4. (doi:10.1016/j.cub.2021.06.070) 34302740

[59] Teunissen N , Kingma SA, Hall ML, Hidalgo Aranzamendi N, Komdeur J, Peters A. 2018 More than kin: subordinates foster strong bonds with relatives and potential mates in a social bird. Behav. Ecol. 29, 1316–1324. (doi:10.1093/beheco/ary120)

[60] Eastwood JR , Hall ML, Teunissen N, Kingma SA, Aranzamendi NH, Fan M, Roast M, Verhulst S, Peters A. 2019 Early-life telomere length predicts lifespan and lifetime reproductive success in a wild bird. Mol. Ecol. 28, 1127–1137. (doi:10.1111/mec.15002) 30592345

[61] Andreasson F , Nord A, Nilsson J-Å. 2020 Age differences in night-time metabolic rate and body temperature in a small passerine. J. Comp. Physiol. B 190, 349–359. (doi:10.1007/s00360-020-01266-5) 32095837

[62] Fan M , Teunissen N, Hall ML, Hidalgo Aranzamendi N, Kingma SA, Roast M, Delhey K, Peters A. 2018 From ornament to armament or loss of function? Breeding plumage acquisition in a genetically monogamous bird. J. Anim. Ecol. 87, 1274–1285. (doi:10.1111/1365-2656.12855) 29943467

[63] Haave-Audet E , Besson AA, Nakagawa S, Mathot KJ. 2022 Differences in resource acquisition, not allocation, mediate the relationship between behaviour and fitness: a systematic review and meta-analysis. Biol. Rev. 97, 708–731. (doi:10.1111/brv.12819) 34859575

[64] Lim JN , Senior AM, Nakagawa S. 2014 Heterogeneity in individual quality and reproductive trade-offs within species. Evolution 68, 2306–2318. (doi:10.1111/evo.12446) 24820133

[65] Lindström J . 1999 Early development and fitness in birds and mammals. Trends Ecol. Evol. 14, 343–348. (doi:10.1016/S0169-5347(99)01639-0) 10441307

[66] Ton R , Boner W, Raveh S, Monaghan P, Griffith SC. 2023 Effects of heat waves on telomere dynamics and parental brooding effort in nestlings of the zebra finch (*Taeniopygia castanotis*) transitioning from ectothermy to endothermy. Mol. Ecol. 32, 4911–4920. (doi:10.1111/mec.17064) 37395529

[67] Friesen CR , Wapstra E, Olsson M. 2022 Of telomeres and temperature: measuring thermal effects on telomeres in ectothermic animals. Mol. Ecol. 31, 6069–6086. (doi:10.1111/mec.16154) 34448287

[68] Dupoué A . 2022 Lizards from warm and declining populations are born with extremely short telomeres. Proc. Natl Acad. Sci. USA 119, e2201371119. (doi:10.1073/pnas.2201371119) PMC9388115 35939680

[69] Axelsson J , Wapstra E, Miller E, Rollings N, Olsson M. 2020 Contrasting seasonal patterns of telomere dynamics in response to environmental conditions in the ectothermic sand lizard, *Lacerta agilis*. Sci. Rep. 10, 182. (doi:10.1038/s41598-019-57084-5) PMC6957525 31932620

[70] Zhang Q , Han X-Z, Burraco P, Hao X, Teng L-W, Liu Z-S, Zhang F-S, Du W-G. 2023 Telomere length, oxidative stress and their links with growth and survival in a lizard facing climate warming. Sci. Total Env. 891, 164 424. (doi:10.1016/j.scitotenv.2023.164424) 37236462

[71] Fitzpatrick LJ , Olsson M, Parsley LM, Pauliny A, Pinfold TL, Pirtle T, While GM, Wapstra E. 2019 Temperature and telomeres: thermal treatment influences telomere dynamics through a complex interplay of cellular processes in a cold-climate skink. Oecologia 191, 767–776. (doi:10.1007/s00442-019-04530-w) 31620874

[72] Seeker LA *et al.* 2021 Telomere attrition rates are associated with weather conditions and predict productive lifespan in dairy cattle. Sci. Rep. 11, 5589. (doi:10.1038/s41598-021-84984-2) PMC7970942 33692400

[73] Foley NM , Petit EJ, Brazier T, Finarelli JA, Hughes GM, Touzalin F, Puechmaille SJ, Teeling EC. 2020 Drivers of longitudinal telomere dynamics in a long-lived bat species, *Myotis myotis*. Mol. Ecol. 29, 2963–2977. (doi:10.1111/mec.15395) 32105386

[74] Simide R , Angelier F, Gaillard S, Stier A. 2016 Age and heat stress as determinants of telomere length in a long-lived fish, the Siberian sturgeon. Physiol. Biochem. Zool. 89, 441–447. (doi:10.1086/687378) 27617363

[75] Urban MC . 2024 Climate change extinctions. Science 386, 1123–1128. (doi:10.1126/science.adp4461) 39636977

[76] Wiens JJ , Zelinka J. 2024 How many species will Earth lose to climate change? Glob. Change Biol. 30, e17125. (doi:10.1111/gcb.17125) 38273487

[77] Williams BA , Watson JEM, Beyer HL, Grantham HS, Simmonds JS, Alvarez SJ, Venter O, Strassburg BBN, Runting RK. 2022 Global drivers of change across tropical savannah ecosystems and insights into their management and conservation. Biol. Conserv. 276, 109786. (doi:10.1016/j.biocon.2022.109786)

[78] Woinarski JCZ , Brock C, Armstrong M, Hempel C, Cheal D, Brennan K. 2000 Bird distribution in riparian vegetation in the extensive natural landscape of Australia's tropical savanna: a broad-scale survey and analysis of a distributional data base. J. Biogeogr. 27, 843–868. (doi:10.1046/j.1365-2699.2000.00439.x)

[79] Reside AE , VanDerWal JJ, Kutt AS, Perkins GC. 2010 Weather, not climate, defines distributions of vagile bird species. PLoS ONE 5, e13569. (doi:10.1371/journal.pone.0013569) PMC2962630 21042575

[80] Teunissen N , McAlpine H, Cameron SF, Murphy BP, Peters A. 2023 Low- and high-intensity fire in the riparian savanna: demographic impacts in an avian model species and implications for ecological fire management. J. Appl. Ecol. 60, 2449–2458. (doi:10.1111/1365-2664.14463)

[81] van Doorn A , Woinarski JCZ, Werner PA. 2015 Livestock grazing affects habitat quality and persistence of the threatened purple-crowned fairy-wren *Malurus coronatus* in the Victoria River District, Northern Territory, Australia. Emu 115, 302–308. (doi:10.1071/MU14073)

[82] Reside AE , VanDerWal J, Garnett ST, Kutt AS. 2016 Vulnerability of Australian tropical savanna birds to climate change. Austral. Ecol. 41, 106–116. (doi:10.1111/aec.12304)

[83] Eastwood JR *et al.* 2026 Data from: Pervasive sublethal impacts of heat exposure revealed by telomere dynamics. (doi:10.5061/dryad.3r2280gwj) PMC13577806 42744327

[84] Eastwood JR *et al.* 2026 Supplementary material from: Pervasive sublethal impacts of heat exposure revealed by telomere dynamics. Figshare. (doi:10.6084/m9.figshare.c.8646249) PMC13577806 42744327

