# Supplementary material for: Pervasive sublethal impacts of heat exposure revealed by telomere dynamics
Source: Proc Biol Sci. 2026 Sep 16;293(2079):20252877. doi: 10.1098/rspb.2025.2877 (PMC13577806; doi:10.1098/rspb.2025.2877)
Supplement: Supplementary file 1 [file rspb.2025.2877.zip › rspb20252877_si_001.pdf]

Supplementary Materials for

Pervasive sublethal impacts of heat exposure revealed by telomere dynamics

Justin R. Eastwood *et al.*

*Corresponding author. Email: justin.eastwood@monash.edu

Doi: 10.1098/rspb.2025-2877

This PDF file includes:

Supplementary Materials and Methods
Supplementary References
Figures S1 to S5
Tables S1 to S6

Other Supplementary Materials for this manuscript include the following:

Data S1

Supplementary Materials and Methods

Telomere length model adjustment

The calculation of relative telomere length was identical to the methods previously described [1]. To improve the precision of our telomere estimates, we combined multiple measurements and accounted for unavoidable sources of methodological error using a linear mixed effects model implemented in the *lme4* package [2], but see [3]. We used our complete telomere dataset, consisting of 6566 relative telomere estimates from 2819 samples of 1503 individuals, to obtain precise estimates. Sample ID and DNA ID were included as random terms to account for repeated measures for each capture. As fixed effects, we included the following methodological variables that have been associated with telomere length: storage buffer (Longmire or ethanol) [1], number of days between sampling and DNA extraction (sample storage time) [4], number of days between DNA extraction and telomere qPCR (DNA storage time), and qPCR plate well position [5]. All fixed predictors significantly ($P < 0.05$) affected telomere length (Table S1), thereby justifying their inclusion for improving telomere precision and accuracy. The predicted values for each sample were exported to calculate the change in telomere length (below). We were unable to include plate ID in our linear mixed-effects model due to the imbalanced sampling design within plates, which was study-based (biased towards age demographic, e.g., nestling or adult) or biased towards recent sampling (all ages collected in recent years tended to be assayed together). Regardless, the variation between plates was accounted for directly in our calculation of the relative telomere estimate using a "gold sample" control repeated four times on each plate and qPCR plate has previously explained little variation [1]. Relative telomere length estimates were standardised before and after model adjustments [6].

Supplementary References

1. Eastwood JR, Mulder E, Verhulst S, Peters A. 2018 Increasing the accuracy and precision of relative telomere length estimates by RT qPCR. *Mol. Ecol. Resour.* **18**, 68–78. (doi:10.1111/1755-0998.12711)

2. Bates D, Mächler M, Bolker B, Walker S. 2015 Fitting linear mixed-effects models using lme4. *J. Stat. Softw.* **1**, 1–48. (doi:10.18637/jss.v067.i01)

3. Evans J, Eastwood J, Teunissen N, Verhulst S, Mikheyev A, Peters A. 2026 Mothers Matter Most: Maternal, but Not Paternal, Age and Inbreeding Affect Nestling Telomere Length in a Wild Passerine. *Mol Ecol* **35**, e70437. (doi:10.1111/mec.70437)

4. Precioso M, Molina-Morales M, Dawson DA, Burke TA, Martínez JG. 2022 Effects of long-term ethanol storage of blood samples on the estimation of telomere length. *Evol Ecol* **36**, 915–931. (doi:10.1007/s10682-022-10198-1)

5. Eisenberg DTA, Kuzawa CW, Hayes MG. 2015 Improving qPCR telomere length assays: Controlling for well position effects increases statistical power. *Am. J. Hum. Biol.* **27**, 570–575. (doi:10.1002/ajhb.22690)

6. Verhulst S. 2020 Improving comparability between qPCR-based telomere studies. *Mol. Ecol. Resour.* **20**, 11–13. (doi:10.1111/1755-0998.13114)

Supplementary figures and tables

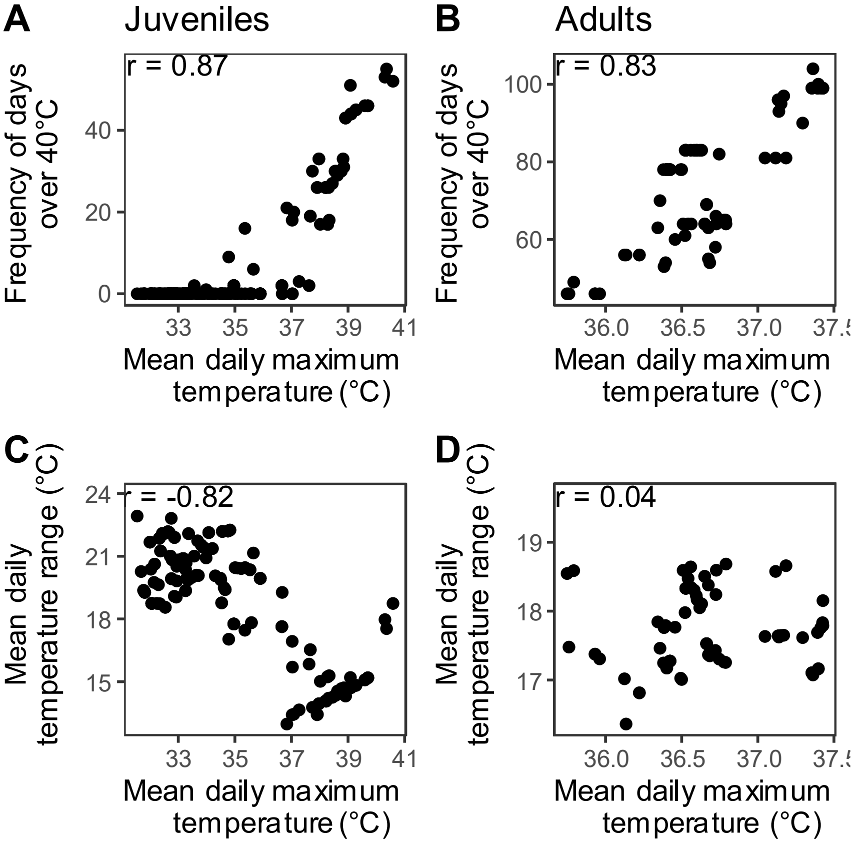

Fig. S1.

The pairwise Pearson correlations (r) between mean daily maximum temperature (°C) and two measures we used to explore temperature extremes, the frequency of days over 40°C (A-B) and mean daily temperature range (mean daily maximum subtracted from minimum; C-D) in our study on telomere attrition in purple-crowned fairy-wrens. A-C shows environmental temperature variables during the juvenile stage, and B-D during the adult stage.

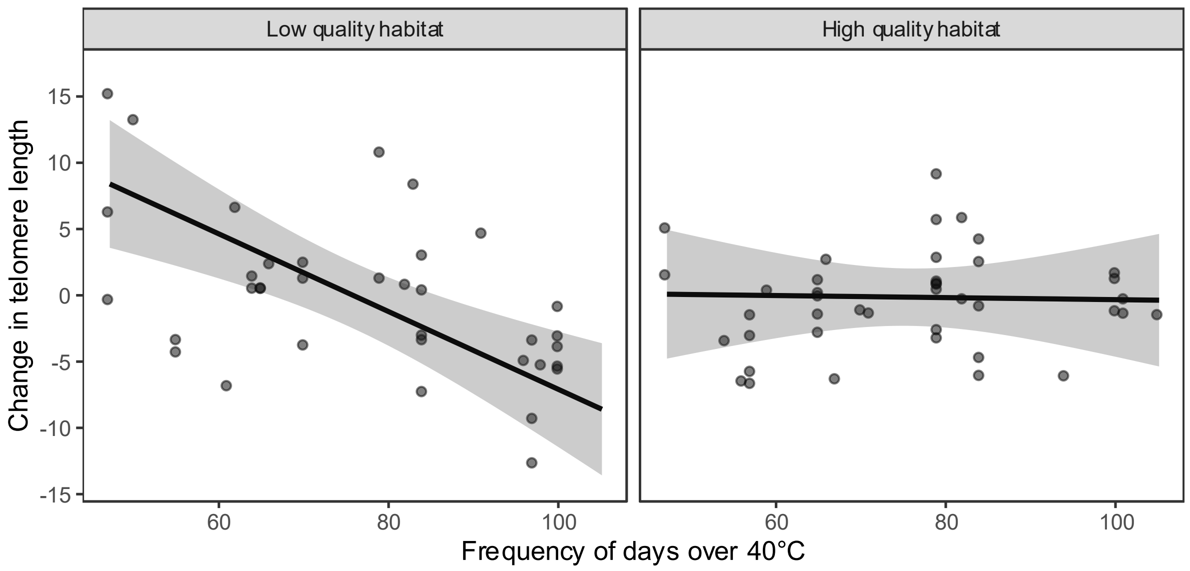

Figure S2.

The density of Pandanus vegetation (habitat quality) modulates the relationship between the frequency of days over 40°C and telomere length change in adult purple-crowned fairy-wrens. Raw data points are presented. Regression lines and confidence intervals were derived from model estimates. We used minimum and maximum habitat quality estimates to visualise the interaction and plotted the raw data points for below and above-average habitat quality values. The frequency of days over 40°C during the adult stage ranged between 46 and 104 days (mean = 75.14, s.d. = 16.52).

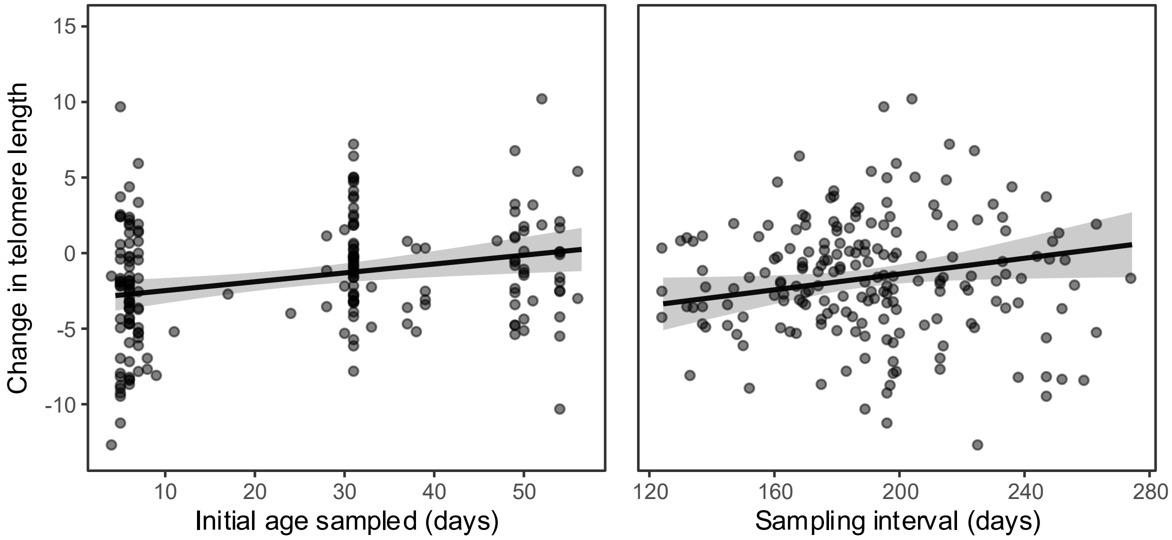

Figure S3.

In juvenile purple-crowned fairy-wrens, we observed increased telomere shortening when individuals were first sampled earlier in life (left; mean = 24.44 days, range 4-56) and when the sampling interval was shorter (right; mean = 190.4 days, range 124-274). Raw data points are presented. Regression lines and confidence intervals were derived from model estimates. It is worth noting that there we observed a mildly non-significant interaction between sampling interval and age initially sampled (Table S4a), which suggests that there is increasingly less telomere shortening when the initial sample was later in life (age first sampled × interval interaction, $p = 0.06$; Table S4a). In other words, telomeres are less dynamic in older juveniles on average.

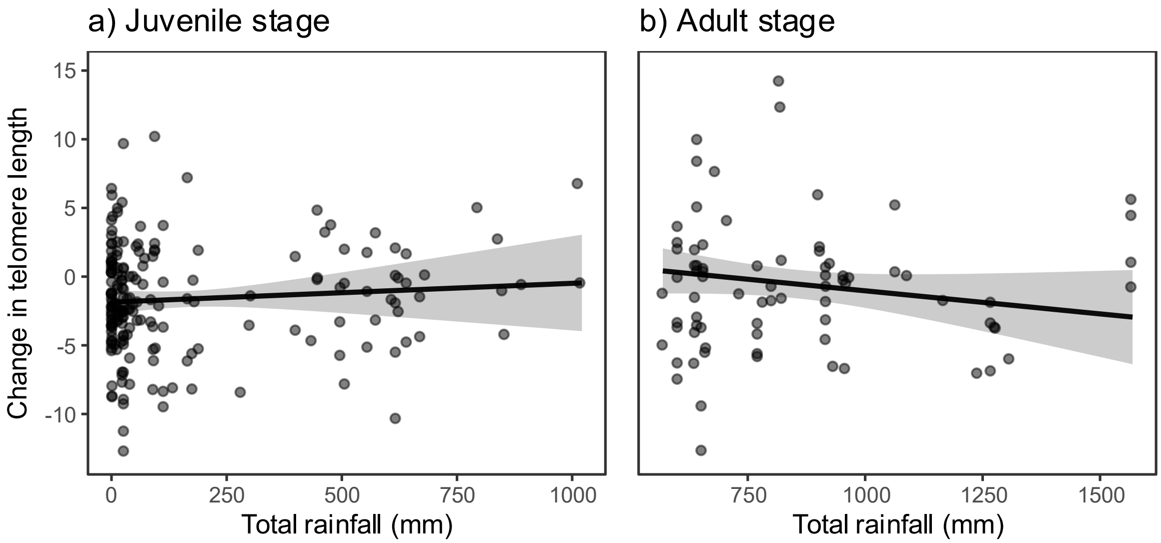

Figure S4.

Rainfall was not a significant predictor of telomere change in the juvenile (a) and adult (b) life stages of purple-crowned fairy-wrens. Raw data points are presented. Regression lines and confidence intervals were derived from model estimates. Total rainfall (mm) over each climate window was mean-centred for analyses (Juvenile stage: mean = 161.14, range = 0-1016.4; Adult stage: mean = 869.14, range = 586.5-1584.3).

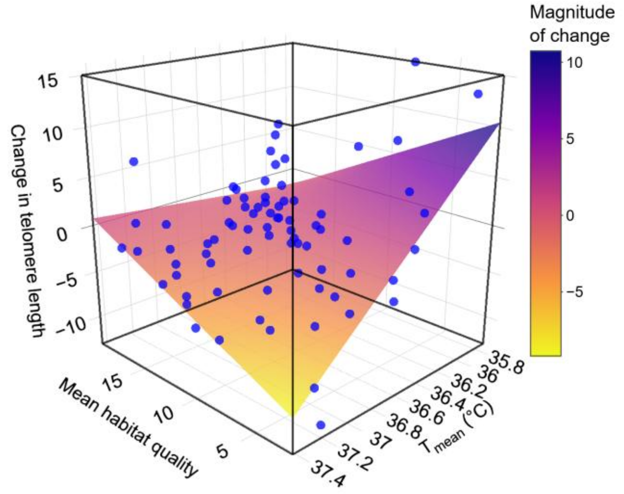

Figure S5.

Adult purple-crowned fairy-wrens are less susceptible (smaller change in telomere length) to hotter conditions when they live in a higher quality habitat (n=77). The change in telomere length is calculated as the difference between 2nd telomere measure and the 1st, so that negative values denote increased telomere attrition. Data points show raw values while the 3D surface area shows model-predicted changes in telomere length.

Table S1.

Type II Wald chi-square tests of significance from the linear mixed effects model adjusting for methodological sources of error on telomere length (n = 6566 relative telomere estimates).

**Methodological source of error**	X^2	**Degrees of freedom**	***P* value**
Blood storage buffer (Longmire or ethanol)	33.1	1	<0.001
Number of days between sampling and DNA extraction (sample storage time)	90.6	1	<0.001
Number of days between DNA extraction and telomere qPCR (DNA storage time)	4.0	1	0.046
qPCR plate well position	288.6	83	<0.001
**Variance components**	σ^2	**Std. dev.**	
DNA ID (n = 3035)	0.350	0.591	
Sample ID (n = 2819)	0.397	0.630	
Residual	0.210	0.458	

Table S2.

A *post hoc* assessment of extreme heat on telomere shortening of purple-crowned fairy-wrens. Here, the final linear mixed-effects models for a) juvenile and b) adult life stages are used, substituting T_{mean} with a measure of extreme temperature, the frequency of days over 40°C (T_{40}). Square brackets indicate the relevant group and reference categories for fixed factors. All continuous variables were mean-centred. Bold text denotes statistical significance ($P < 0.05$).

	**a) Juvenile ΔTL n = 192**				**b) Adult ΔTL n = 77**			
**Fixed effects**	**Estimate (SE)**	**d.f.**	**t**	**p**	**Estimate (SE)**	**d.f.**	**t**	**p**
Intercept	-2.03 (0.51)	138.80	-4.01	<0.001	-0.16 (0.85)	59.50	-0.19	0.85
Individual								
Sex [Female, ref. Male]	0.21 (0.55)	181.12	0.38	0.71	0.65 (1.30)	61.07	0.51	0.62
Sampling interval (days)	0.02 (0.01)	182.29	1.54	0.13	0.01 (0.01)	63.69	1.35	0.18
**Initial age sampled (days)**	**0.07 (0.02)**	**174.95**	**3.20**	**0.002**	-	-	-	-
Social								
Mean group size	0.35 (0.23)	137.63	1.56	0.12	-0.21 (0.45)	64.39	-0.46	0.64
Status [Dom., ref. Help.]	-	-	-	-	-1.56 (1.47)	66.70	-1.06	0.29
Dispersal [yes., ref. no]	-	-	-	-	1.33 (1.33)	60.05	1.00	0.32
Environmental								
Habitat quality (score)	0.03 (0.06)	105.85	0.42	0.68	-0.02 (0.12)	57.95	-0.16	0.87
Natal season [Dry, ref. Wet]	0.83 (0.88)	164.63	0.94	0.35	-	-	-	-
**T_{40}**	-0.05 (0.03)	182.81	-1.55	0.12	**-0.14 (0.05)**	**30.81**	**2.94**	**0.006**
Rainfall (mm)	0.001 (0.002)	178.36	0.30	0.77	-0.01 (0.003)	41.13	-1.95	0.06
Interactions								
**T_{40} × Habitat quality**	-	-	-	-	**0.02 (0.01)**	**58.98**	**2.43**	**0.02**
**Random effects**	**σ^2**	**Std. dev.**			**σ^2**	**Std. dev.**		
Maternal ID	1.12	1.06			3.69	1.92		
Residual	12.97	3.60			17.22	4.15		
**Model fit**	**R^2 marg.**	**R^2 cond.**			**R^2 marg.**	**R^2 cond.**		
	0.10	0.17			0.23	0.36		

Table S3.

A *post hoc* assessment of environmental temperature variability on telomere shortening of purple-crowned fairy-wrens. Here, using the final linear mixed-effects models for a) juvenile and b) adult life stages, T_{mean} was substituted with the mean daily minimum and maximum air temperature difference (T_{range}). Square brackets indicate the relevant group and reference categories for fixed factors. Note that including the interaction between T_{range} and habitat quality in the adult model was not statistically significant and therefore, removed from the model. Square brackets indicate the relevant group and reference categories for fixed factors. All continuous variables were mean-centred. Bold text denotes statistical significance ($P < 0.05$).

	**a) Juvenile ΔTL n = 192**				**b) Adult ΔTL n = 77**			
**Fixed effects**	**Estimate (SE)**	**d.f.**	**t**	**p**	**Estimate (SE)**	**d.f.**	**t**	**p**
Intercept	-1.87 (0.50)	136.86	-3.72	<0.001	-0.76 (0.92)	59.55	-0.82	0.42
Individual								
Sex [Female, ref. Male]	0.28 (0.55)	181.87	0.51	0.61	1.40 (1.41)	60.95	0.99	0.32
Sampling interval (days)	0.02 (0.01)	179.45	1.47	0.14	0.01 (0.01)	63.72	0.79	0.44
**Initial age sampled (days)**	**0.06 (0.02)**	**173.57**	**3.06**	**0.003**	-	-	-	-
Social								
Mean group size	0.38 (0.23)	137.01	1.70	0.09	-0.23 (0.51)	58.89	-0.45	0.65
Status [Dom., ref. Help.]	-	-	-	-	-1.72 (1.68)	69.85	-1.02	0.31
Dispersal [yes., ref. no]	-	-	-	-	1.68 (1.46)	60.97	1.15	0.25
Environmental								
Habitat quality (score)	-0.02 (0.06)	105.36	0.25	0.80	-0.01 (0.13)	56.24	-0.03	0.97
Natal season [Dry, ref. Wet]	0.27 (0.83)	163.79	0.34	0.74	-	-	-	-
T_{range} (°C)	-0.04 (0.21)	116.76	-0.19	0.85	0.81 (1.13)	61.80	0.71	0.48
Rainfall (mm)	-0.002 (0.003)	174.96	-0.65	0.52	-0.0002 (0.002)	50.13	0.06	0.95
Interactions								
T_{range} × Habitat quality	-	-	-	-	-	-	-	-
**Random effects**	**σ^2**	**Std. dev.**			**σ^2**	**Std. dev.**		
Maternal ID	0.91	0.96			1.55	1.25		
Residual	13.32	3.65			23.79	4.88		
**Model fit**	**R^2 marg.**	**R^2 cond.**			**R^2 marg.**	**R^2 cond.**		
	0.09	0.14			0.05	0.11		

Table S4.

The final linear mixed-effects model shows that the mean maximum daily temperature (T_{mean}, °C) negatively affects a) juvenile and b) adult purple-crowned fairy-wrens. Square brackets indicate the relevant group and reference categories for fixed factors. All continuous variables were mean-centred. Bold text denotes $P < 0.05$.

	a) Juvenile ΔTL n = 192				b) Adult ΔTL n = 77			
**Fixed effects**	**Estimate (SE)**	**d.f.**	**t**	**p**	**Estimate (SE)**	**d.f.**	**t**	**p**
Intercept	-2.25 (0.52)	135.24	-4.31	<0.001	-0.11 (0.84)	59.49	-0.13	0.90
Individual								
Sex [Female, ref. Male]	0.23 (0.54)	180.05	0.42	0.67	-0.18 (1.30)	59.07	-0.14	0.89
**Sampling interval (days)**	**0.02 (0.01)**	**182.14**	**2.09**	**0.04**	0.01 (0.01)	61.58	0.84	0.41
**Initial age sampled (days)**	**0.06 (0.02)**	**175.91**	**2.82**	**0.01**	-	-	-	-
Social								
Mean group size	0.30 (0.23)	142.06	1.29	0.20	-0.27 (0.44)	64.65	-0.61	0.55
Status [Dom., ref. Help.]	-	-	-	-	-1.12 (1.44)	66.93	-0.78	0.44
Dispersal [yes., ref. no]	-	-	-	-	1.95 (1.30)	56.49	1.51	0.14
Environmental								
Habitat quality (score)	0.05 (0.06)	110.20	0.79	0.43	-0.001 (0.12)	57.49	-0.01	0.995
Natal season [Dry, ref. Wet]	1.38 (0.92)	160.40	1.51	0.13	-	-	-	-
**T_{mean} (°C)**	**-0.50 (0.21)**	**168.07**	**-2.38**	**0.02**	**-4.54 (1.37)**	**34.48**	**-3.31**	**0.002**
Rainfall (mm)	0.001 (0.002)	182.79	0.67	0.50	-0.004 (0.002)	47.05	-1.48	0.15
Interactions								
**T_{mean} × Habitat quality**	-	-	-	-	**0.79 (0.27)**	**60.37**	**2.96**	**0.004**
**Random effects**	**σ^2**	**Std. dev.**			**σ^2**	**Std. dev.**		
Maternal ID	1.51	1.23			3.12	1.77		
Residual	12.43	3.53			16.66	4.08		
**Model fit**	**R^2 marg.**	**R^2 cond.**			**R^2 marg.**	**R^2 cond.**		
	0.11	0.21			0.26	0.38		

Table S5.

The global linear mixed-effects models, including all interactions, for a) juvenile and b) adult life stages of purple-crowned fairy-wrens. T_{mean} refers to the mean maximum daily air temperature (°C). Square brackets indicate the relevant group and reference categories for fixed factors. All continuous variables were mean-centred. Bold text denotes $P < 0.05$.

	**a) Juvenile ΔTL n = 192**				**b) Adulthood ΔTL n = 77**			
**Fixed effects**	**Estimate (SE)**	**d.f.**	**t**	**p**	**Estimate (SE)**	**d.f.**	**t**	**p**
Intercept	-1.15 (0.85)	146.12	-1.36	0.18	0.62 (1.00)	64.08	0.62	0.54
Individual								
Sex [Female, ref. Male]	0.28 (0.54)	178.16	0.52	0.61	-0.33 (1.32)	64.44	-0.25	0.80
**Sampling interval (days)**	**0.03 (0.01)**	**173.99**	**2.41**	**0.02**	0.01 (0.01)	62.38	0.90	0.37
**Initial age sampled (days)**	**0.08 (0.02)**	**174.57**	**3.46**	**<0.001**	-	-	-	-
Social								
Mean group size	0.21 (0.23)	126.81	0.90	0.37	-0.14 (0.47)	64.91	-0.30	0.77
Status [Dom., ref. Help.]	-	-	-	-	-1.58 (1.46)	64.49	-1.09	0.28
Dispersal [yes., ref. no]	-	-	-	-	1.54 (1.34)	58.27	1.15	0.26
Environmental								
Habitat quality (score)	0.05 (0.06)	105.97	0.80	0.43	-0.05 (0.12)	63.84	-0.37	0.71
Natal season [Dry, ref. Wet]	1.13 (0.91)	147.39	1.23	0.22	-	-	-	-
**T_{mean} (°C)**	**-0.62 (0.23)**	**174.25**	**-2.75**	**0.01**	**-4.55 (1.45)**	**41.94**	**-3.14**	**0.003**
Rainfall (mm)	0.01 (0.004)	178.99	1.34	0.18	-0.001 (0.004)	62.05	-0.32	0.75
Interactions								
Sampling interval × Initial age sampled	0.001 (0.001)	168.09	1.91	0.06	-	-	-	-
T_{mean} × Rainfall	-0.002 (0.001)	176.06	-1.24	0.22	0.006 (0.007)	63.11	0.87	0.39
**T_{mean} × Habitat quality**	-0.01 (0.02)	178.13	-0.59	0.56	**0.73 (0.28)**	**65.00**	**2.63**	**0.01**
T_{mean} × Mean group size	-0.14 (0.09)	176.62	-1.54	0.13	-1.32 (0.97)	64.96	-1.37	0.18
**Random effects**	**σ^2**	**Std. dev.**			**σ^2**	**Std. dev.**		
Maternal ID	0.86	0.93			6.52	2.55		
Territory ID	0	0			0	0		
Natal year	0	0			-	-		
Residual	12.71	3.57			13.91	3.73		
**Model fit**	**R^2 marg.**	**R^2 cond.**			**R^2 marg.**	**R^2 cond.**		
	0.14	0.20			0.29	0.52		

Table S6.

Results of the linear mixed-effects model examining whether initial telomere length is negatively associated with habitat quality in adult-stage purple-crowned fairy-wrens (n = 77). Territory ID was included as a random intercept. The capture year was initially included as a random effect but was removed from the final model because it explained <0.0001% variance. Square brackets indicate the group and reference category for categorical fixed effects. All continuous predictor variables were mean-centred and standardised (z-transformed). Bold text indicates statistically significant effects ($P < 0.05$).

Fixed effects	Estimate (SE)	d.f.	t	p
Intercept	-0.14 (0.17)	48.78	-0.81	0.42
**Initial telomere length**	**-0.11 (0.04)**	**34.96**	**-2.57**	**0.01**
Age at capture	-0.10 (0.05)	38.16	-1.82	0.08
Sex [Female, ref. Male]	0.16 (0.10)	33.02	1.53	0.14
**Mass at capture**	**-0.15 (0.06)**	**37.68**	**-2.52**	**0.02**
Tarsus at capture	0.08 (0.05)	34.16	1.55	0.13
Season [Wet, ref. Dry]	0.06 (0.09)	34.15	0.71	0.48
**Random effects**	**σ^2**	**Std. dev.**	**ICC**	
Territory identity (n = 41)	1.00	1.00	0.95	
Residual	0.05	0.23		

Data S1. (separate file)

All data used in this study are available at Dryad https://doi.org/10.5061/dryad.3r2280gwj
